# Why Records?

**Published:** 1918-12

**Authors:** 


### WHY RECORDS?

For action! Yes! To put backbone into plans. To give toughness of fibre for argument. To be the starting point of improvement. Without facts we flounder. We have floundered, begging the experience of past wars to help us solve the miracles of this.

Not for historians are the files kept, but for reasoning men to turn to, to keep them from the easy path of theory and prediction, to give them a basis for action. Why make a correct diagnosis? Because a man's life depends on it, or his ease of mind and body demand appropriate treatment, and because there is honesty and credit in it. Why change the diagnosis before or after autopsy? Because a clinical lie rests no easier than a financial one.

Picture to yourself that Cosmos, the army, a patient with his obstetrical difficulties at birth, his puling stage of colics and febriculas, his rashes, his coughs. The schooling and the college is past, and here is the splendid man in full maturity in our hands. Never was he more precious to others or to us, and here we plant him against a man's job.

Shall we cease our daily, almost hourly gauges of his fitness? Is it his head which is strained to bursting with endeavor and emotion? Are his feet lame and cold, holding back the forward sweep of troops? Has he brought with him the seeds of disease from childhood, the worms, plasmodia, bacteria of other climes and races? Is he wilting in the close caverns made fetid by human crowding? What is it that cuts off each fraction of his full possibilities and makes his fighting arm less quick and powerful, his back less straight to carry, his eye less keen to see and judge?

Such are the questions the Chief Surgeon must answer to himself, to his General, to the Staff and to our Nation of parents and children at home.

How can it be done? Has he done it? What use does he make of his facts, if he can get them? Yes, it can be done though it has never been attempted in the A. E. F. way before.

The diagnostician is the cornerstone of the whole structure. His error or his fact are subject to no review, unless perchance the pathologist humbles the clinician as is his wont at the autopsy table. The moment a patient is admitted to a hospital he is listed by the Registrar, and every 24 hours there goes forward from each hospital and from every other medical formation with hospital facilities, a list of the admissions, changes of diagnosis, deaths and discharges. Received at the Chief Surgeon's office, a strange transformation attacks this medley of names, diseases, organizations, hospitals and dates. The devil of modern efficiency seizes upon this interminable sheet and transforms it into cards, embroidered with small holes, and the coding process is undertaken which translates the sick roll into fodder for the tabulating machines. Before the day is done, the three or four thousand individual reports of new cases, deaths and dispositions are all listed, sorted, tabulated and recorded so that the actual loss of man power from any disease by organization, or arm of service, by hospital, section or date, can be, at but a moment's notice, provided for the next day's use. A turn of the electric light switch and the Hollerith sorting machines with almost human intelligence, but with far more than human infallibity, gobble up a pile of cards and re-arrange them so that they tell a truthful story of the day's experience, and give a dramatic record of that unending struggle between man and his environment, through which so many of our troops are temporarily or permanently put out of the battle line.

Do you wish to know the proportion of days lost from the itch, from pneumonia, from gunshot wounds of the head, from flat-feet? Or are you interested in the competition between the Air service and the Engineers in diseases of the ear? Or is it a comparison between the S. O. S. and the combat divisions in incidence of dysentery which brings you to the office? Is it necessary to provide hospital service for special groups of diseases? For all these questions, recourse must be had at once to the records of the Sick and Wounded Department of the Chief Surgeon's office, which can give you not only the incidence of disease by days or any multiple thereof, but the time which must be allowed for absence from duty for the different diseases, and the expected disposition by return to duty, discharge, death or otherwise disposed of. Does the General Staff wish an answer as to the result of disciplinary measures instituted to control venereal disease in one section or another, among one group of troops or another? Here is their answer promptly and completely, if the diagnostician has been accurate and the reporting officer has transmitted the information correctly and promptly.

No physician in the army, however much of an individualist he may be in his own personal philosophy, can shirk his responsibility to his fellowmen by failing to record accurately the diagnosis and medical information, which his professional training puts him in the position of acquiring and transmitting. The Fact Factory is one of the liveliest parts of the medical department and full of possibilities for immediate use and future students.

